# Facing the music: three issues in current research on singing and aphasia

**DOI:** 10.3389/fpsyg.2014.01033

**Published:** 2014-09-23

**Authors:** Benjamin Stahl, Sonja A. Kotz

**Affiliations:** ^1^Brain Language Laboratory, Department of Philosophy and HumanitiesFreie Universität Berlin, Berlin, Germany; ^2^Max Planck Institute for Human Cognitive and Brain SciencesLeipzig, Germany; ^3^School of Psychological Sciences, University of ManchesterManchester, UK

**Keywords:** left-hemisphere stroke, speech-language pathology, non-fluent aphasia, apraxia of speech, Melodic Intonation Therapy, singing, rhythmic pacing, formulaic language

Left-hemisphere stroke patients suffering from speech and language disorders are often able to sing entire pieces of text fluently. This finding has inspired a number of music-based rehabilitation programs, most notable among them a treatment known as Melodic Intonation Therapy (Albert et al., 1973). According to the inventors of the treatment, singing should promote a transfer of language function from left frontotemporal neural networks to their preserved right-hemisphere homologues. Although singing indeed engages right frontotemporal areas (Callan et al., 2006; Özdemir et al., 2006), it does not seem to induce a transfer of language function from the left to the right hemisphere (Belin et al., 1996; Jungblut et al., 2014). Nonetheless, several studies confirmed the promising role of singing (Mills, 1904; Gerstmann, 1964; Keith and Aronson, 1975; Tomaino, 2010) and the overall efficacy of Melodic Intonation Therapy (Van der Meulen et al., 2014).

Using an analytic research approach, two recent experiments explored whether singing, rhythmic pacing, and lyric type have an immediate effect on syllable production (Stahl et al., 2011) or a lasting effect on some aspects of speech and language recovery (Stahl et al., 2013). Contrary to earlier reports, the results did not indicate a short- or long-term advantage of singing over rhythmic speech in persons with non-fluent aphasia and apraxia of speech. Rather, the results revealed that lyric type may be of great importance. Conversational speech formulas—such as “good morning,” “everything alright?” or “I'm fine”—yielded higher rates of correctly produced syllables than novel word sequences, whether they were sung or rhythmically spoken. Moreover, the sung and the spoken training of a few selected speech formulas proved to facilitate the production of these phrases.

We readily acknowledge that increasing the variety of phrases may lead to generalized effects in therapy, while singing in syllable-timed languages such as French possibly adds to the level of rhythmicity in a particular way (cf. Schmidt-Kassow et al., 2011; Zumbansen et al., 2014). Still, this does not fully explain the range of seemingly contradictory findings in the literature. In our opinion paper, we would like to address three issues in current research on singing and aphasia: articulatory tempo, clinical research designs, and formulaic language resources. We believe that these issues may account for some of the major discrepancies between past reports.

## Issue 1: articulatory tempo

Singing slows down articulatory tempo. This, in turn, has been found to benefit syllable production in patients with speech and language disorders (Beukelman and Yorkston, 1977; Laughlin et al., 1979; Pilon et al., 1998; Hustad et al., 2003). Although the interaction of articulatory tempo and syllable production is often useful in therapy, it may lead to problems in clinical research, as illustrated in a cross-sectional study (Racette et al., 2006). Eight patients were singing and speaking novel lyrics in two conditions: alone (solo word production), and together with a vocal playback (choral word production). During solo word production, the number of intelligible words was independent of whether the patients were singing or speaking. During choral word production, the number of intelligible words was generally higher, with more sung than spoken words articulated correctly. Based on these results, one may conclude that choral singing facilitates word production in aphasic patients.

Taking a closer look at the experimental design, however, some details of the study are worth noting. The sung playback voice produced words only half as fast as the spoken playback voice. This difference also affected the patients' actual performance. During solo word production, the patients were relatively free to choose their preferred articulatory tempo (mean [*M*] duration of sung syllables: *M* = 572 ms; spoken syllables: *M* = 494 ms; Δ = 78 ms; cf. Racette et al., 2006). During choral word production, the patients adapted to the articulatory tempo of the sung and spoken vocal playbacks (sung syllables: *M* = 696 ms; spoken syllables: *M* = 426 ms; Δ = 270 ms). Hence, the mean difference in syllable duration between singing and speaking (Δ) was 3.5 times larger during choral word production than during solo word production.

This comparison reveals that the patients had more time to articulate well when they were singing to vocal playback. Without a doubt, the reported results suggest a facilitating effect of choral word production, whether sung or spoken. However, the results do not necessarily indicate a benefit from choral singing. What seems like a choral singing effect may actually arise from differences in articulatory tempo. Using a similar research design, but controlling for articulatory tempo, a later study did not confirm an effect of choral singing over choral speech in 17 patients (Stahl et al., 2011). The consistent duration of sung and spoken syllables may be one of the reasons for this contrary finding. In summary, the control of articulatory tempo appears to be crucial in studies that seek to determine how different forms of vocal expression affect syllable production in patients with speech and language disorders.

## Issue 2: clinical research designs

Another issue concerns clinical studies that address the underlying mechanisms of music-based aphasia therapy. One seminal treatment study investigated the efficacy of Melodic Intonation Therapy (Schlaug et al., 2008). A language test indicated that Melodic Intonation Therapy was more effective than a non-melodic control treatment in 2 chronic aphasic patients. Although the results may not generalize to a larger clinical population, they nonetheless provide evidence for the efficacy of Melodic Intonation Therapy in a group of chronic aphasic patients. However, it is worth considering whether or not the results offer insight into the efficacy of any particular therapeutic element included in the program—such as singing—and its underlying neural mechanisms, as revealed by structural and functional imaging.

It is important to note that Melodic Intonation Therapy includes various forms of vocal expression and multi-modal feedback: singing minor thirds; rhythmic speech with exaggerated prosody (sprechgesang); tactile rhythmic stimulation via hand tapping; choral word and phrase production; solo word and phrase repetition; auditory cueing of initial word and phrase syllables; and so on (cf. Helm-Estabrooks et al., 1989). Given the number of stimulating elements used in Melodic Intonation Therapy, it is challenging to assess the contribution of each element to the efficacy of the entire program. This problem becomes especially apparent if one compares the composition of non-melodic control treatments and Melodic Intonation Therapy in clinical studies.

In the study mentioned above, the non-melodic control treatment did not include singing, rhythmic sprechgesang, and rhythmic hand tapping, whereas Melodic Intonation Therapy did (cf. Schlaug et al., 2008). That is, the treatments did not only differ in singing, but also in other aspects of vocal expression and sensorimotor feedback. Consequently, the results do not necessarily support the clinical efficacy of singing and its underlying neural mechanisms. What seems like a benefit from singing may actually be the effect of rhythm, prosody, tactile stimulation, or any of their combinations. Considering this range of possible interpretations, it is not a contradiction that a recent experiment did not confirm a long-term effect of singing over rhythmic speech in 10 chronic aphasic patients (Stahl et al., 2013). In summary, the interpretation of clinical results depends on whether the underlying research design focuses on music-based aphasia therapy as an entire program or on its specific mechanisms.

## Issue 3: formulaic language resources

One reason for the success of music-based aphasia therapy may be its use of common phrases. The original manual of Melodic Intonation Therapy proposes phrases such as “I love you,” “how are you?” or “thank you” at the lower proficiency level of the program (Helm-Estabrooks et al., 1989). The phrases are stereotyped in form, tied to social context and, therefore, fall into the category of formulaic language (Van Lancker Sidtis, 2004). This fact is critical: according to present knowledge, the production of formulaic language engages bilateral neural networks including right frontotemporal areas, the right basal ganglia and, possibly, the right cerebellum (Hughlings-Jackson, 1878; Speedie et al., 1993; Ackermann et al., 1998; Van Lancker Sidtis et al., 2003; Sidtis et al., 2009). The degree of right-hemisphere lateralization seems to be strongest for pragmatically oriented formulaic language: pause fillers, discourse elements, and conversational speech formulas (Van Lancker Sidtis and Postman, 2006). It is intriguing to consider what these results may imply for clinical practice.

Formulaic expressions may be viewed as a valuable language resource in patients with left-hemisphere lesions. Recent evidence suggests that standard speech-language therapy facilitates newly created utterances, while the massive repetition of conversational speech formulas leads to long-term progress in the production of trained phrases (Stahl et al., 2013). Eight out of ten patients were able to establish their own individual formulaic repertoire to communicate some basic needs in daily life. Moreover, the therapeutic use of conversational speech formulas may have a motivating and rewarding effect, most notably in patients with extended left frontotemporal and subcortical lesions. Patients often report feeling competent if they are able to produce a few phrases correctly. Finally, it is conceivable that even non-formulaic expressions become part of the formulaic repertoire by means of massive repetition: patients may eventually retrieve the trained phrases as a coherent unit from the mental lexicon, engaging bilateral or right-hemisphere neural networks (cf. Wolf et al., 2014).

The crucial role of formulaic language in music-based aphasia therapy challenges the interpretation of structural and functional neuroimaging data. Until recently, the sensitivity of right frontotemporal areas to Melodic Intonation Therapy has been presumed to result from the neural plasticity of non-formulaic language (Schlaug et al., 2008, 2009; Vines et al., 2011). However, the imaging data may also depend on the plasticity of right-hemisphere neural networks that support the production of formulaic language. What seems like a melody-mediated transfer of language function from the left to the right hemisphere may actually be the use of right-hemisphere language resources. Behavioral evidence for this claim comes from the finding that music-based aphasia therapy benefits the production of conversational speech formulas, irrespective of whether the patients are singing (Stahl et al., 2013). In summary, future research needs to consider the possible interplay of music-based aphasia therapy and right-hemisphere neural networks engaged in the production of formulaic language.

## Outlook on future research

There is no doubt that music-based aphasia therapy—including Melodic Intonation Therapy—is a promising treatment for several types of speech and language disorders. The rhythmic elements of the program may help to overcome deficits in motor planning, commonly found in persons with apraxia of speech. The training of formulaic and non-formulaic phrases may indirectly compensate for communicative difficulties associated with agrammatism, as is the case in persons with Broca's aphasia. The repetitive character of the program as well as the focus on a limited formulaic repertoire may be suitable to alleviate severe expressive and receptive symptoms, typically observed in persons with global aphasia. In other words, the use of melody-based aphasia therapy seems appropriate for a number of speech and language disorders.

To this day, research on music-based aphasia therapy has addressed the overall efficacy of current rehabilitation programs (holistic approach) and some of their underlying mechanisms (analytic approach). Drawing on the findings from holistic approaches, the long-term goal of analytic research is to tailor future rehabilitation programs to the individual needs of the patients. This may increase the efficacy of the treatment to a considerable extent. One may argue that analytic research on music-based aphasia therapy is taking a reductionist view, for example, by disentangling the close relationship between melody and rhythm (cf. Merrett et al., 2014). Until now, however, analytic research on music-based aphasia therapy has actually compared singing—that is, the *combined* use of melody and rhythm—with other forms of vocal expression, including rhythmic speech. The available data are, therefore, consistent with the idea that rhythmicity is naturally inherent in singing.

Still, analytic research on music-based aphasia therapy should not overshadow the valuable contribution of holistic approaches. We believe that both holistic and analytic approaches are needed and usually depend on each other. Many clinical hypotheses are derived from analytic research and then tested in holistic designs, and vice versa. Acting in concert, holistic and analytic approaches may help to improve the quality of research in the field as well as the individual efficacy of music-based aphasia therapy.

### Conflict of interest statement

The authors declare that the research was conducted in the absence of any commercial or financial relationships that could be construed as a potential conflict of interest.
